# Endoscopic vs. Microscopic Resection of Sellar Lesions—A Matched Analysis of Clinical and Socioeconomic Outcomes

**DOI:** 10.3389/fsurg.2017.00033

**Published:** 2017-06-22

**Authors:** Tej D. Azad, Yu-Jin Lee, Daniel Vail, Anand Veeravagu, Peter H. Hwang, John K. Ratliff, Gordon Li

**Affiliations:** ^1^Department of Neurosurgery, Stanford University School of Medicine, Stanford, CA, United States; ^2^Department of Otolaryngology, Stanford University School of Medicine, Stanford, CA, United States

**Keywords:** endoscopy, microscopy, pituitary, sellar lesions, complication rate

## Abstract

**Background:**

Direct comparisons of microscopic and endoscopic resection of sellar lesions are scarce, with conflicting reports of cost and clinical outcome advantages.

**Objective:**

To determine if the proposed benefits of endoscopic resection are realized on a population level.

**Methods:**

We performed a matched cohort study of 9,670 adult patients in the MarketScan database who underwent either endoscopic or microscopic surgery for sellar lesions. Coarsened matching was applied to estimate the effects of surgical approach on complication rates, length of stay (LOS), costs, and likelihood of postoperative radiation.

**Results:**

We found that LOS, readmission, and revision rates did not differ significantly between approaches. The overall complication rate was higher for endoscopy (47% compared to 39%, OR 1.37, 95% CI 1.22–1.53). Endoscopic approach was associated with greater risk of neurological complications (OR 1.32, 95% CI 1.11–1.55), diabetes insipidus (OR 1.65, 95% CI 1.37–2.00), and cerebrospinal fluid rhinorrhea (OR 1.83, 95% CI 1.07–3.13) compared to the microscopic approach. Although the total index payment was higher for patients receiving endoscopic resection ($32,959 compared to $29,977 for microscopic resection), there was no difference in long-term payments. Endoscopic surgery was associated with decreased likelihood of receiving post-resection stereotactic radiosurgery (OR 0.67, 95% CI 0.49–0.90) and intensity-modulated radiation therapy (OR 0.78, 95% CI 0.65–0.93).

**Conclusion:**

Our results suggest that the transition from a microscopic to endoscopic approach to sellar lesions must be subject to careful evaluation. Although there are evident advantages to transsphenoidal endoscopy, our analysis suggests that the benefits of the endoscopic approach are yet to be materialized.

## Introduction

Traditionally, sellar lesions such as pituitary adenomas, craniopharyngiomas, and Rathke’s cleft cysts were resected using a transsphenoidal approach with microscopy (1). However, since the introduction of endoscopic instruments and technique described by Jankowski et al. in 1992, many institutions have reported a gradual transition to the endoscopic approach (2, 3). The microscopic approach requires a nasal speculum to fracture the nasal septum and a sphenoidotomy to facilitate access to the anterior skull base. Endoscopic surgery may use one or both nostrils for access following a posterior septectomy. The endoscopic view is noted to provide a wider degree of freedom and improved visual access to the sella, although limitations related to lens cleaning and “sword fighting” with instruments have been acknowledged (4). The microscopic approach provides three-dimensional visualization and surgeon familiarity with the operating microscope (5).

The body of evidence comparing these two techniques is largely comprised of retrospective case series or cohort studies from single institutions (6–12). While individual studies report differences between the two approaches in complication rates, including cerebrospinal fluid (CSF) leak, temporary and permanent diabetes insipidus, hypopituitarism, meningitis, carotid artery injury, and visual and rhinologic complications, a consistent benefit regarding complication rates has not been observed in systematic reviews and meta-analyses (13–16). However, many studies report that endoscopic approach is associated with a shorter length of hospital stay (14, 17–20) and may be more cost-effective in the long term based on modeling (21, 22) as well as in a single-institution series (23).

The objectives of this study were to compare the microscopic and endoscopic approaches to assess (1) 30-day complication rates; (2) length of stay (LOS), readmission, and revision rate; (3) costs; and (4) likelihood of post-resection radiation therapy. To date, the comparison of endoscopic vs. microscopic approach to sellar lesions has not been conducted using a large database that contains a representative sample of the United States population.

## Materials and Methods

### Data Source

We performed a retrospective observational administrative database study of 9,670 adult patients who underwent either endoscopic or microscopic surgery for sellar lesions in the United States from 2007 to 2014. We used claims data from the Truven Health Analytics MarketScan Commercial Claims and Encounters and Medicare Supplemental and Coordination of Benefits databases, which include data from >100 payers and use specific patient identifiers for longitudinal patient tracking of claims, billing, and payment history. The longitudinal characteristic of this database enabled assessment of patient comorbidities and complications both before and after the index procedure. The MarketScan database also allows tracking of patient health-care expenditures for the entirety of their private insurance coverage.

### Cohort Definition

We queried the MarketScan database for patients who received either microscopic or endoscopic resection of sellar lesions. We utilized Current Procedural Terminology (CPT) codes to define the cohort (CPT 61548, microscopic approach; CPT 62165, neuroendoscopic approach). Patients who underwent open surgery, who were younger than 18 years, and who had concurrent microscopic and endoscopic CPT codes were excluded from this analysis (Figure S1 in Supplementary Material). International Classification of Diseases, Ninth Revision, Clinical Modification (ICD-9-CM) codes were used to characterize surgical indications and comorbidities. Subsequent revision procedures, complications, and in-hospital mortality were determined for all patients. The ICD-9 and CPT codes utilized in our study are detailed in Table S1 in Supplementary Material.

### Statistical Analysis

We used matched and unmatched regression models to estimate the effects of an endoscopic surgical approach on postoperative complication rates, mortality, length of hospital stay, readmission rates, surgical revision rates, costs of care, and extent of tumor resection. Patients with missing data for a variable of interest were excluded from the analysis. We used logistic regression to estimate models with binary outcomes (presence of various complications within 30 days of surgery, whether patient was discharged home after surgery, whether patient was readmitted to the hospital within 30 days of surgery, whether patient required a surgical revision, extent of tumor resection, and mortality), and used linear regression for continuous outcomes (length of hospital stay and total costs of care). All regression models controlled for the following covariates, which were considered likely to be associated with the outcomes of our models: patient age, gender, year of procedure, region of the country where the patient resides, and relevant comorbidities diagnosed prior to surgery (Cushing’s disease, acromegaly, hypertension, congestive heart failure, chronic obstructive pulmonary disorder, diabetes, obesity, past drug abuse, psychiatric illness, depression, electrolyte imbalance, hypothyroidism, and anemia).

We ran all regression models on our full sample of 9,670 patients. As a sensitivity analysis, we also ran the same models on a matched subset of our sample to correct for covariate imbalance in the full sample. This matched subset included 6,577 patients. We used coarsened exact matching (CEM) to create an analytic sample in which all covariates were distributed similarly among patients who received endoscopy and patients who received microscopic surgery. CEM is similar to propensity score matching, but it allows the analyst to specify a minimum degree of covariate balance and reduces model dependence and estimation error compared to propensity score matching (24). Similar results in the matched and unmatched models would indicate that covariate imbalance was not substantial enough to bias the results of our unmatched models.

## Results

### Patient Characteristics and Trends in Surgical Approach for Sellar Lesions

**Figure 1:**
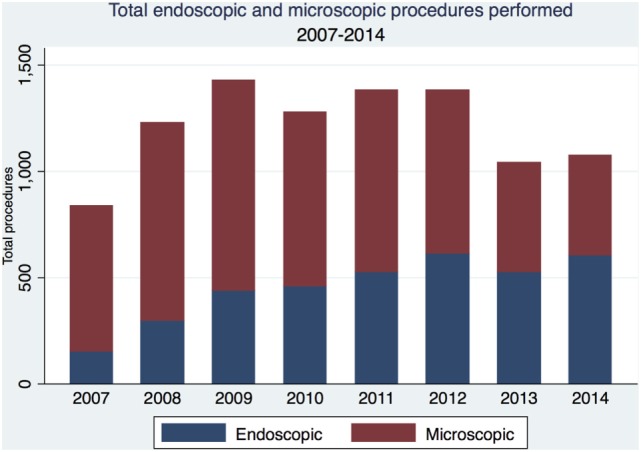
Trends in the utilization of the microscopic and endoscopic approaches from 2007 to 2014.

From 2007 to 2014, we identified 3,621 patients (54.5% female, median age 50 years) who received endoscopic surgery and 6,049 patients (52.7% female, median age 50 years) who received microsurgery for sellar lesions. The most common primary surgical indications, as indicated by the preoperative ICD-9-CM codes, for both surgical approaches were identical, with over 80% of operations performed for benign neoplastic lesions of the pituitary gland and craniopharyngeal duct (ICD-9 code 227.3). Length of inpatient stay (3.5 days for endoscopic surgery vs. 3.6 days for microsurgery) did not differ significantly between these cohorts, while follow-up length was significantly longer among patients who received microsurgery (877 vs. 719 days for endoscopic surgical patients, *p* < 0.01). The distribution of demographic characteristics between endoscopic and microscopic approaches is given in Table 1. Coarsened matching allowed us to model cohorts balanced for patient demographics and comorbidities, also reflected in Table 1. We further observed an increase in total procedures during the duration of this study, with endoscopic surgery comprising a greater proportion of total procedures over time (Figure 1).

**Table 1:** Demographics and comorbidities of the unmatched and matched cohorts.

	Unmatched (*N* = 9,670)			Matched (*N* = 6,577)		
	Microscopy	Endoscopy	*p*-Value	Microscopy	Endoscopy	*p*-Value
Age	49.2	48.8	0.18	48.5	48.3	0.76
Female	3,200 (52.9%)	1,977 (54.6%)	0.11	48.0%	48.0%	1
Cushing’s disease	508 (8.4%)	308 (8.5%)	0.87	5.0%	5.0%	1
Acromegaly	405 (6.7%)	333 (9.2%)	<0.01	6.0%	6.0%	1
Hypertension	2,704 (44.7%)	1,673 (46.2%)	0.16	40.0%	40.0%	1
CHF	163 (2.7%)	87 (2.4%)	0.49	4.0%	4.0%	1
COPD	756 (12.5%)	529 (14.6%)	<0.01	7.0%	7.0%	1
Diabetes	1,222 (20.2%)	753 (20.8%)	0.44	14.0%	14.0%	1
Obesity	665 (11.0%)	503 (13.9%)	<0.01	6.0%	6.0%	1
Drug abuse	53 (0.88%)	34 (0.94%)	0.76	1.0%	1.0%	1
Psychiatric illness	357 (5.9%)	253 (7.0%)	0.03	1.0%	1.0%	1
Depression	538 (8.9%)	355 (9.8%)	0.15	3.0%	3.0%	1
Electrolyte imbalance	502 (8.3%)	331 (9.1%)	0.17	3.0%	3.0%	1
Hypothyroidism	1,210 (20.0%)	865 (23.9%)	<0.01	17.0%	17.0%	1
Deficiency anemia	726 (12.0%)	500 (13.8%)	0.01	7.0%	7.0%	1
Blood loss anemia	53 (0.88%)	47 (1.3%)	0.06	1.0%	1.0%	1

*Values in unmatched cohort are frequencies and percentages while values in the matched cohort are predicted probabilities*.

### Thirty-Day Complication Rates

**Figure 2:**
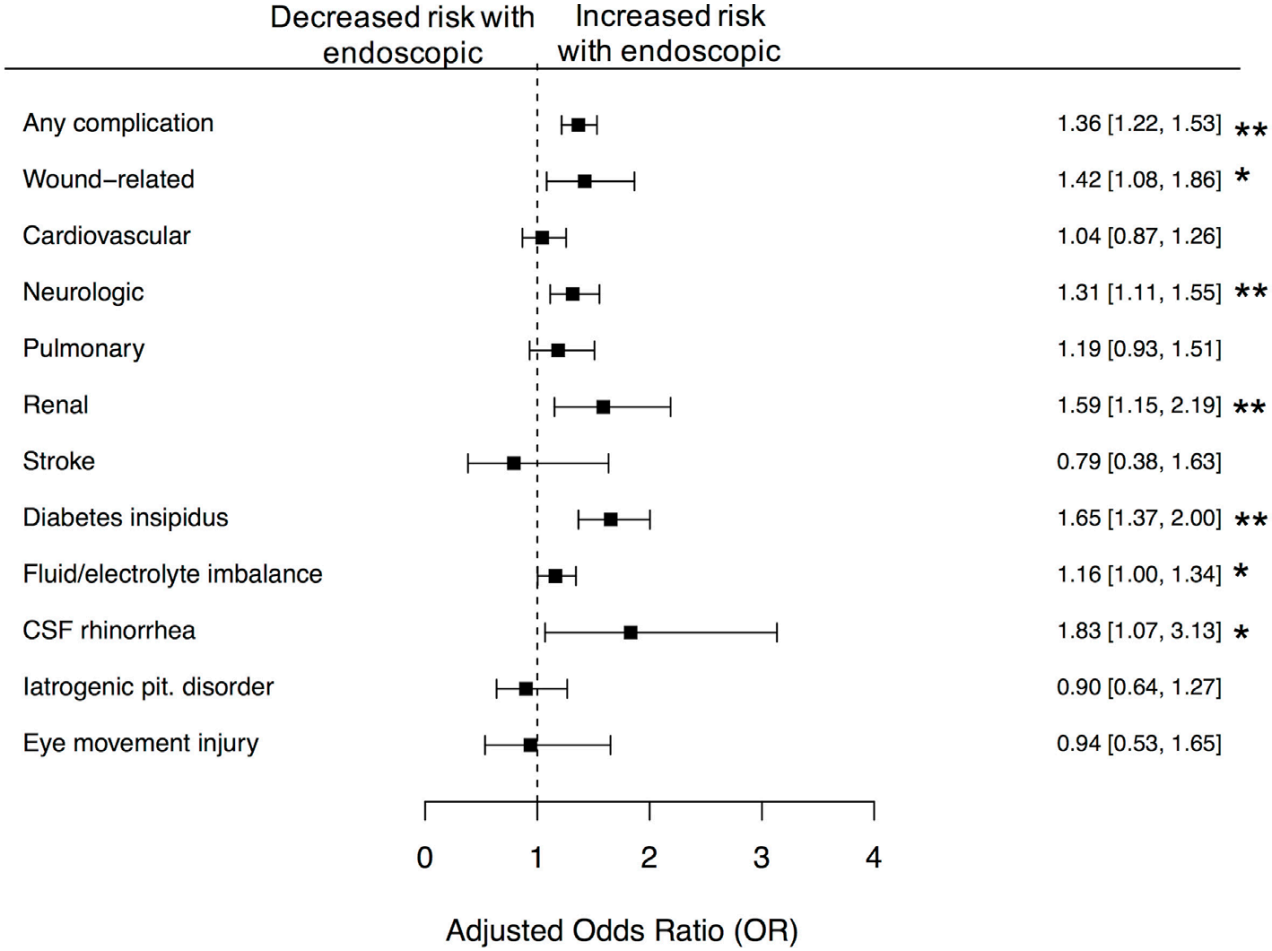
Differences in 30-day complications between microscopic and endoscopic approaches, following adjustment with CEM. Following coarsened exact matching (CEM), we observed significantly increased risk of any complication (*p* < 0.01), wound-related complications (*p* < 0.05), neurologic complications (*p* < 0.01), renal complications (*p* < 0.01), diabetes insipidus (*p* < 0.01), fluid and electrolyte imbalance (*p* < 0.05), and CSF rhinorrhea (*p* < 0.05), following receipt of endoscopic approach. Error bars indicate 95% CI; dashed line, no difference (OR = 1.0).

After matching the endoscopic and microscopic cohorts, we observed a greater risk of any complication when the endoscopic approach was used at a rate of 47% compared with 39% for microscopic approach (OR 1.37, 95% CI 1.22–1.53). We further found that patients receiving endoscopic surgery were at significantly greater risk of wound-related complications at a rate of 5.2 vs. 3.7% (OR 1.42, 95% CI 1.08–1.86), neurologic complications at a rate of 15 vs. 12% (OR 1.32, 95% CI 1.11–1.55), renal complications at a rate of 3.8 vs. 2.4% (OR 1.59, 95% CI 1.15–2.19), diabetes insipidus at a rate of 12 vs. 7.9% (OR 1.65, 95% CI 1.37–2.00), fluid electrolyte imbalance at a rate of 19.4 vs. 17.2% (OR 1.16, 95% CI 1.00–1.34), and cerebrospinal fluid (CSF) rhinorrhea at a rate of 1.7 vs. 0.9% (OR 1.83, 95% CI 1.07–3.13). We did not observe any significant differences between the two approaches for eye movement injury (1.1% in endoscopic vs. 1.2% in microscopic) or iatrogenic pituitary disorder (2.6% in endoscopic vs. 2.9% in microscopic) (Figure 2).

### Quality and Payment Characteristics

We found that 30-day all-cause readmission rates at 28% for both endoscopic and microscopic approaches (OR 0.98, 95% CI 0.86–1.12), rate of discharge home at a rate of 94% for both approaches (OR 1.03, 95% CI 0.82–1.29), and inpatient mortality at a rate of 0.2% for endoscopic compared to 0.4% for microscopic approach (OR 0.63, 95% CI 0.17–2.36) were comparable between the two approaches in the matched cohort. While we observed that endoscopic surgery was protective for receipt of revision procedure (OR 0.73, 95% CI 0.57–0.92) in the unmatched cohort, this difference did not reach statistical significance after controlling for differences in demographics and comorbidities between those who received endoscopic surgery and those who did not (OR 0.77, 95% CI 0.58–1.34) (Table 2).

**Table 2:** Key quality indicators for microscopic and endoscopic resection of sellar lesions.

	Predicted probability (CI) for microscopic resection	Predicted probability (CI) for endoscopic resection	OR (CI)
30-day readmission	0.283 (0.266–0.300)	0.280 (0.261–0.298)	0.984 (0.864–1.120)
Revision	0.046 (0.038–0.054)	0.036 (0.028–0.044)	0.774 (0.578–1.038)
Discharged home	0.939 (0.930–0.948)	0.940 (0.931–0.950)	1.029 (0.819–1.293)
Mortality	0.004 (0.000–0.007)	0.002 (0.000–0.005)	0.626 (0.166–2.355)
Length of stay, days	3.539 (3.365–3.714)	3.489 (3.356–3.622)	−0.051 (−0.252–0.151)^⊥^

*Following adjustment using CEM, predicted probabilities for each outcome were generated for both groups and odds ratios were calculated*.

*^⊥^Coefficient, not OR*.

*OR, odds ratio; CI, 95% confidence interval; N, sample size*.

In the matched cohorts, we found significantly higher total index payments for patients receiving endoscopic resection at $32,959 compared to $29,977 for microscopic resection (mean increase of $2,982.78, 95% CI 1,316.28–4,649.29). Notably, we observed no difference in either 90- or 180-day payments between the groups (Table 3). The majority of these higher index payments went to hospitals (hospital payments were $2,508.87 higher among endoscopy patients, *p* < 0.01) rather than to physicians (no significant difference).

**Table 3:** Comparative payments between microscopic and endoscopic approaches during the initial inpatient admission and at 90 and 180 days.

	Predicted costs (CI) for microscopic resection	Predicted costs (CI) for endoscopic resection	Betas (CI)
Inpatient costs	$29,977 ($28,957–30,996)	$32,959 ($31,549–34,370)	$2,982.78 ($1,316.28–4,649.29)**
90-day costs	$9,198 ($8,259–10,137)	$9,619 ($8,623–10,614)	$420.97 (−$954.80–1,796.75)
180-day costs	$14,708 ($13,426–15,990)	$15,052 ($13,687–16,416)	$344.12 (−$1,527.20–2,215.45)

**p < 0.01.

*CI, 95% confidence interval*.

### Radiation Post-Resection

**Figure 3:**
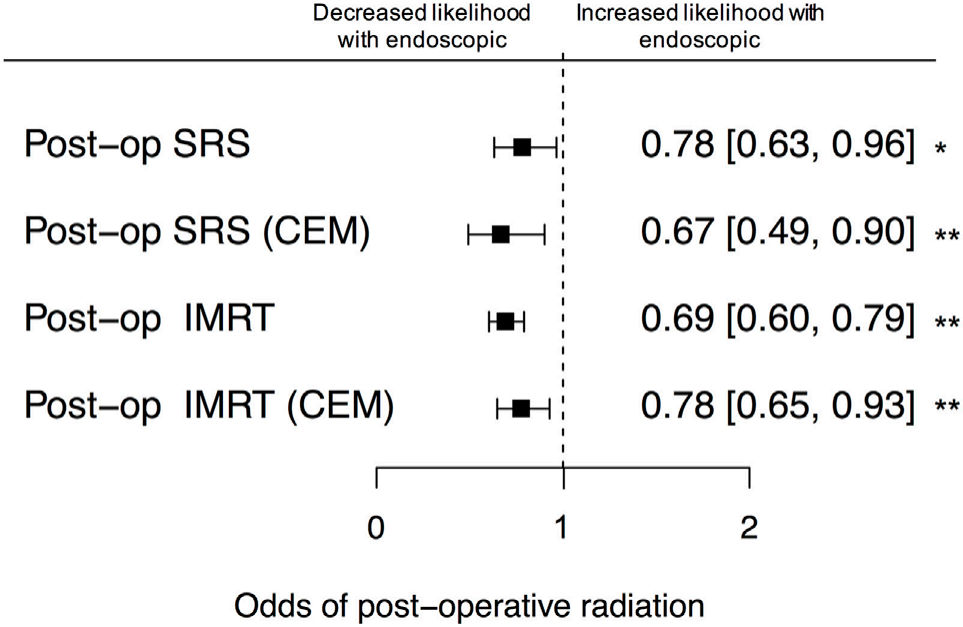
Likelihood of post-resection stereotactic radiosurgery (SRS) and intensity-modulated radiation therapy (IMRT). There was a lower unadjusted odds of receiving either SRS or IMRT following endoscopic resection, relative to microscopic resection. After coarsened exact matching (CEM), these differences were preserved and statistically significant (*p* < 0.01). Error bars indicate 95% CIs; dashed line, no difference (OR = 1.0).

We observed an overall decrease in likelihood of receiving post-resection radiation after endoscopic surgery relative to microscopic surgery in both the unmatched and matched cohorts (Figure 3). The endoscopic approach was predicted to decrease the likelihood of receiving post-resection stereotactic radiosurgery (SRS) in the unmatched (OR 0.76, 95% CI 0.61–0.95) and matched cohorts (OR 0.67, 95% CI 0.49–0.90). Similarly, the endoscopic approach was predicted to decrease likelihood of receiving post-resection intensity-modulated radiation therapy in the unmatched (OR 0.79, 95% CI 0.69–0.91) and matched cohorts (OR 0.78, 95% CI 0.65–0.93).

## Discussion

Based on existing literature of single-institution studies and systematic reviews, we expected no significant differences between the complication rates when comparing microscopic and endoscopic approach to resection of sellar lesions using the MarketScan claims database. However, our results provide additional and unexpected insight into the recent practice of the two techniques from 2007 to 2014 in a large representative sample of the general United States population that allows rigorous statistical analysis with strict matching criteria.

Our endoscopic (*n* = 3,621) and microscopic (*n* = 6,049) cohorts were overall similar, including primary indications for surgery. This suggests that both approaches are currently considered viable in the same clinical context. Neither approach seemed to prefer one indication over the other, although this may be due to the high prevalence of benign pituitary lesions as well as the non-specific diagnostic code in ICD-9, “227.3 Benign neoplasm of pituitary gland and craniopharyngeal duct.” We saw an increase in endoscopic approach over the eight years of study duration. This result is consistent with the literature of single-center studies that report institutional transition from microscopic to endoscopic approach.

Complication and mortality rate are major ways of assessing whether one procedure is preferable to the other. While endoscopic approach was protective in the context of inpatient mortality rates, this result was not significant at the alpha level of 0.05. Endoscopic cases had 37% higher odds of any 30-day complication compared to microscopic cases as well as higher odds for specific complications including diabetes insipidus, electrolyte imbalance, CSF rhinorrhea, wound-related, neurologic, and renal complications. This is unexpected, given that existing studies suggest overall no difference between the two approaches. However, most single-institution studies were conducted in high-volume endoscopic centers while our study includes cases from all levels of care. Interestingly, although there were higher odds of complications among the endoscopic cases, the endoscopic approach was observed to have decreased rates of revision procedure in the unmatched comparison. However, this result was not statistically significant in the strictly matched cohort. The higher rate of complications along with decreased rate of revision suggests a more aggressive resection in the endoscopic approach.

Since transsphenoidal endoscopic approach is a relatively new technique compared to the traditional gold standard of the microscopic approach, we examined whether there was a training effect that may play a role in the higher complication rate observed among endoscopic cases. However, the complication rate for the endoscopic approach remains higher than microscopic throughout every year of our data and does not appear to be trending downward at a faster rate than the microscopic approach. To further evaluate complication rate on an individual basis, we anticipate further studies that compare complication rates among individual surgeons stratified by volume based on several studies examining the learning curve associated with transsphenoidal endoscopic surgery (3, 25). It is possible that our finding of a higher complication rate in endoscopic resection is due to surgeon experience and/or volume. This result has been suggested in previous Nationwide Inpatient Sample studies (1, 26).

While this is important to bear in mind, the observation itself is important for surgeons and patients to be aware of. The single-institution studies in the literature that describe outcomes of endoscopy are from early-adopters and often from high-volume centers. Our finding is that the advantages of endoscopic resection have not yet materialized on a population level.

Additionally, there was a 21% reduction in odds for stroke complication in endoscopic cases compared to microscopic cases in our matched cohort, although this finding was not significant at an alpha level of 0.05. Carotid injury was a rare complication in our study with only five occurrences in endoscopic cases and two occurrences in microscopic cases. Given the small sample size, we could not analyze whether this difference was statistically significant.

Length of hospital stay, 30-day readmission rate, and rate of discharge home were similar for both endoscopic and microscopic cases, which was surprising because previous studies had shown the endoscopic approach to have shorter LOS. One major advantage of this dataset is the longitudinal follow-up of patients based on unique identifiers. Based on this, we could characterize the duration of follow up, which was significantly longer among microscopic cases. This may be due to differences in preference of the duration of follow-up between endoscopic vs. microscopic surgeons, as increased complication rates do not seem to correlate with the increased length of follow-up. Although the length of hospital stay was shorter in the endoscopic cohort (3.5 vs. 3.6 days), endoscopic cases had higher total index payment by $2,982.78. Given that the 90-day and 180-day payments were similar, the increased cost from longer duration of follow-up among microscopic cases may balance the higher index cost for endoscopic cases.

The likelihood of receiving post-resection radiation in the endoscopic group was overall decreased relative to the microscopic group in both stereotactic radiosurgery and intensity-modulated radiation therapy. The decreased likelihood in the endoscopic group may suggest that the endoscopic group had less residual tumor and more complete resection. This may also explain the increased complication rates in the endoscopic group, suggesting that endoscopic approach is more aggressive, undergoes more complete resection, and requires less post-resection radiation.

One important limitation of our study is the inability to analyze complication rates stratified by tumor size and extent, which may confound the actual complication rates attributable to each technique. Many groups employ an endoscopic approach for anatomically extensive tumors not amenable to microscopic resection and this difference may underlie the observed differences in complication rates. However, this is an inherent limitation of the claims data since there are no ICD-9 or CPT codes that provide indication with respect to the size or extent of the tumor.

## Conclusion

Taken together, our results suggest that the transition from a microscopic to endoscopic approach to sellar lesions must be subject to careful evaluation. While there are evident advantages to transsphenoidal endoscopy, our analysis suggests that the benefits of the endoscopic approach are yet to be materialized.

## Author Contributions

TA, AV, PH, JR, and GL developed the concept for the study. TA and DV carried out statistical analysis. TA, YJL, DV, AV, PH, JR, and GL contributed to the drafting of the paper and editing of the final manuscript.

## Conflict of Interest Statement

The authors declare that the research was conducted in the absence of any commercial or financial relationships that could be construed as a potential conflict of interest.

## Supplementary Material

Figure S1 CONSORT diagram.

Table S1 Compendium of ICD9/CPT codes used in the study.
