# Supplementary figures and images for: Endoscopic vs. Microscopic Resection of Sellar Lesions—A Matched Analysis of Clinical and Socioeconomic Outcomes
Source: Front Surg. 2017 Jun 22;4:33. doi: 10.3389/fsurg.2017.00033 (PMC5479879; doi:10.3389/fsurg.2017.00033)

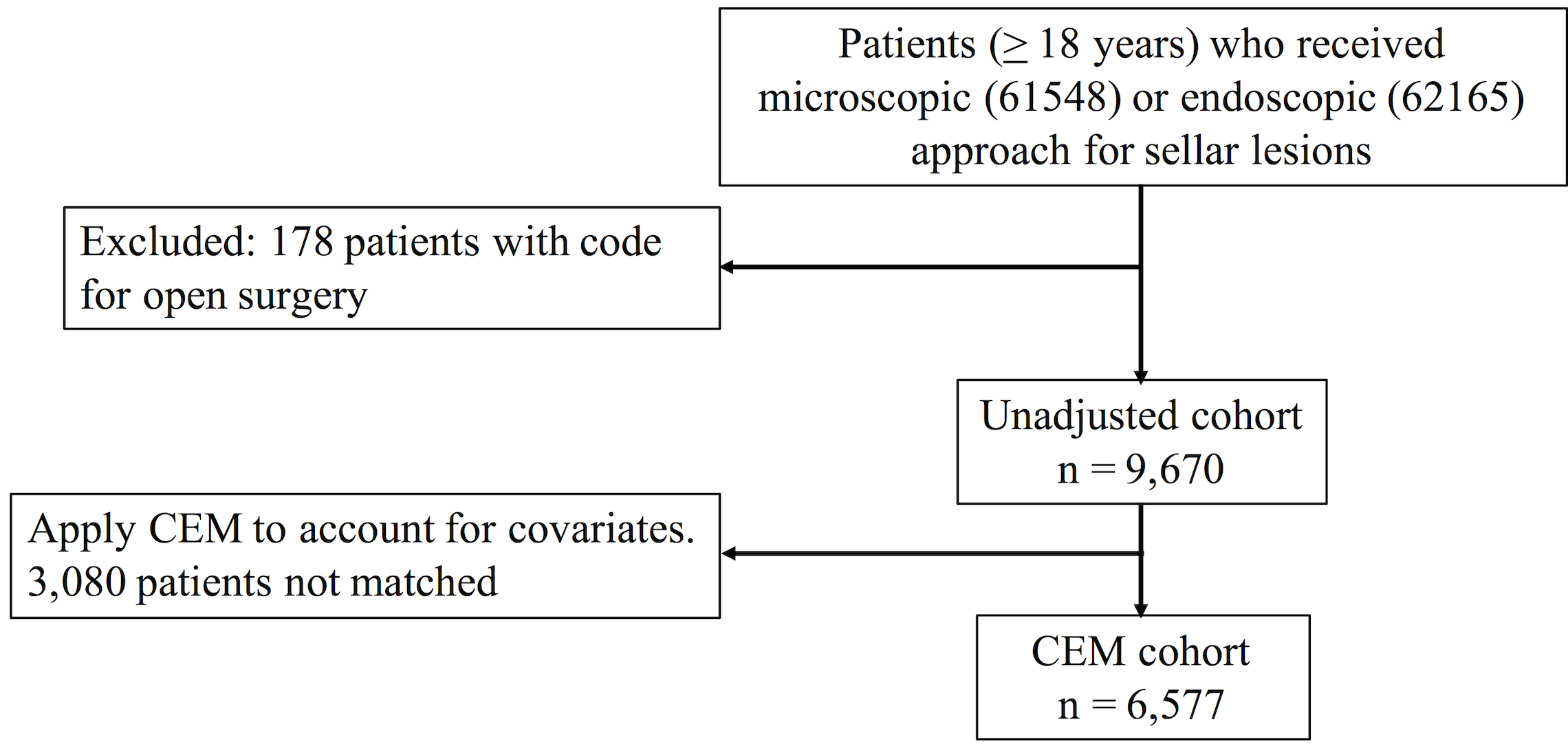

Supplement: Table S1 — Compendium of ICD9/CPT codes used in the study. [file Image_1.TIFF]
