# Supplementary material for: Endoscopic vs. Microscopic Resection of Sellar Lesions—A Matched Analysis of Clinical and Socioeconomic Outcomes
Source: Front Surg. 2017 Jun 22;4:33. doi: 10.3389/fsurg.2017.00033 (PMC5479879; doi:10.3389/fsurg.2017.00033)
Supplement: Supplementary file 2 [file Table_1.DOCX]

		Definition (code type)	ICD-9/CPT Codes
	Cohort Definition Codes	Sellar resection	
		Microscopic approach (CPT)	61548
		Endoscopic approach (CPT)	62165
		Diagnosis	
		Malignant sellar neoplasm (ICD-9)	194.3
		Benign sellar neoplasm (ICD-9)	227.3
		Sellar neoplasm of uncertain behavior (ICD-9)	237.0
		Acromegaly (ICD-9)	253.0
		Cushing's disease (ICD-9)	255.0
		Other and unspecified anterior pituitary hyperfunction (ICD-9)	253.1
	Exclusion Criteria	Open approach for sellar resection (CPT)	61546
Covariates		COPD (ICD-9)	416.8, 416.9, 490–505, 506.4, 508.1, 508.8
		Drug abuse (ICD-9)	292.0, 292.8-292.9, 304, 305.2-305.9, 648.3
		Psychoses (ICD-9)	295-298, 299.1
		Depression (ICD-9)	300.4, 301.12, 309.00, 309.1, 311
		Fluid/electrolyte imbalance (ICD-9)	276.0-9
		Deficiency anemia (ICD-9)	280, 285.2, 285.9
		Blood loss anemia (ICD-9)	280.0, 648.2
		Hypothyroidism (ICD-9)	243-244
	Cardiac	CHF (ICD-9)	398.91, 402.01, 402.11, 402.91, 404.01, 404.03, 404.11, 404.13, 404.91, 404.93, 425.4–425.9, 428
		Hypertension (ICD-9)	401, 402-405, 642, 437.2
		MI / Ischemic Heart Disease (ICD-9)	410, 412, 998.0, 9971, 411, 429.7
	Metabolic	Diabetes (ICD-9)	250.0–250.7
		Obesity (ICD-9)	278.0, 649.1, 793.91, V853, V854, V8554
	Complications	Infection (ICD-9)	998.5, 998.0, 999.3
		Infection (CPT)	10060, 10140, 10180, 12020, 12021, 20005, 21501, 22010, 22015
		Wound dehiscence or disruption (ICD-9)	998.3
		Wound hematoma (ICD-9)	998.1
		Other wound complication (ICD-9)	998.3, 998.81, 998.83, 998.4
		Cardiovascular (ICD-9)	427, 426.1, 426, 426.3, 426.4, 426.5, 426.6, 426.7, 426.8, 453.0, 453.4, 453.8, 453.2, 453.3, 451, 453.4, 453.8, 453.2, 453.1, 453.9, 410, 412, 998.0, 997.1, 411, 429.7
		Neurologic (ICD-9)	430, 431, 432, 433, 434, 435, 436, 438.2, 438.3, 438.4, 438.5, 997.0
		Pulmonary (ICD-9)	415.1, 997.3, 518.4, 518.5, 518.7, 518.81, 518.82, 518.83, 518.84, 518.89, 519.1
		Renal (ICD-9)	997.5, 584.5, 584.6, 584.7, 584.8, 584.9
		Stroke (ICD-9)	997.02
		Diabetes insipidus (ICD-9)	253.5
		Fluid/electrolyte imbalance (ICD-9)	276.0-9
		CSF rhinorrhea (ICD-9)	349.81
		Iatrogenic pituitary disorder (ICD-9)	253.7
		Eye movement injury (ICD-9)	368.2, 374.30, 374.31, 378.50-9
